# Combining transradial access and sheathless femoral access for complex iliac artery chronic total occlusions

**DOI:** 10.1186/s42155-022-00334-x

**Published:** 2022-10-24

**Authors:** Naoki Hayakawa, Satoshi Kodera, Keisuke Takanashi, Shinya Ichihara, Satoshi Hirano, Masataka Arakawa, Yasunori Inoguchi, Junji Kanda

**Affiliations:** 1 Department of Cardiovascular Medicine, Asahi General Hospital, I-1326 Asahi, Chiba, 289-2511 Japan; 2 Department of Cardiovascular Medicine, The University of Tokyo Hospital, Tokyo, Japan

**Keywords:** Transradial intervention, Iliac artery, Chronic total occlusion, Endovascular therapy

## Abstract

**Background:**

The transradial approach (TRA) is associated with fewer serious access site-related complications compared with the transfemoral or transbrachial approach. However, TRA has associated problems in complex aortoiliac (AI) lesions, including the procedural difficulty. A bidirectional approach was used combining TRA with a sheathless technique for femoral artery (FA) puncture to treat complex AI lesions, as a minimally-invasive approach. This report describes a representative cases with AI chronic total occlusion in which the combination of TRA and a sheathless technique for FA puncture was useful for guidewire crossing.

**Case presentation:**

Case 1 was a 71-year-old man with intermittent claudication (IC). Control angiography showed total occlusion of the left common iliac artery (CIA) ostium to the distal external iliac artery (EIA). Guidewire externalization was achieved by combining TRA using a 6Fr guiding sheath and a sheathless technique for the left FA. Two nitinol stents were deployed in the CIA to EIA. Case 2 was a 63-year-old man with IC. Control angiography revealed total occlusion of the right CIA ostium to the common femoral artery (CFA) with severe calcification. The antegrade wire could not pass through the CTO lesion because of the calcified CFA occlusion. A 21-G metal needle was used to penetrate the CFA calcification through the distal true lumen of the CFA, and the wire was inserted into the EIA for wire externalization. Three nitinol stents were deployed in the CIA to EIA, and a drug-coated balloon was dilated in the CFA with hemostasis of the distal puncture site. In both cases, the retrograde puncture site was hemostatic during the procedure and postoperative bed rest was not required.

**Conclusions:**

TRA combined with a sheathless technique from the FA has the potential to treat AI complex lesions in a less invasive manner.

## Background

The efficacy of endovascular therapy (EVT) for the aortoiliac (AI) artery is well established, and developments in EVT have led to it becoming one of the first-line treatment strategies for AI occlusive diseases (Soga et al., 2012; Yamauchi et al., 2019). The transfemoral approach (TFA) is the standard approach for EVT. However, this approach can be associated with puncture site-related complications, such as hematoma, pseudoaneurysm, arteriovenous fistula, and retroperitoneal hemorrhage (Ortiz et al., 2014). The transbrachial approach (TBA) has been used as an alternative to the TFA. However, the reported rate of access site-related complications is 14.0% (Treitl et al., 2015). The percutaneous transradial approach (TRA) has been established as a standard approach for percutaneous coronary intervention (PCI) because of fewer serious access site-related complications (Kiemeneij et al., 1997; Valgimigli et al., 2015).

The use of the TRA to the AI artery was reported to have benefits, such as feasibility and fewer complications (Shinozaki et al., 2014; Lorenzoni et al., 2017; Meertens et al., 2018). However, for treatment of complex lesions, including iliac long chronic total occlusion (CTO), the rate of failure with the TRA only or the need to switch to a femoral access site remains high (Lorenzoni et al., 2017). This situation appears to arise because the device lacks adequate backup and the guidewire operability, represented by torque response and the pushing force of the guidewire, is poor. A 6Fr transradial system is now available for EVT. This system allows for completion of a more complex procedure and contains a bare nitinol stent (BNS) in the regular-size lineup (Shinozaki et al., 2019). In this study, a bidirectional approach was used combining a TRA with the TRA system and a sheathless technique for femoral artery (FA) puncture to treat complex iliac CTO, as a less invasive approach. This article describes this technique through representative cases of iliac artery CTO.

## Case presentations

### Case 1

**Fig. 1:**
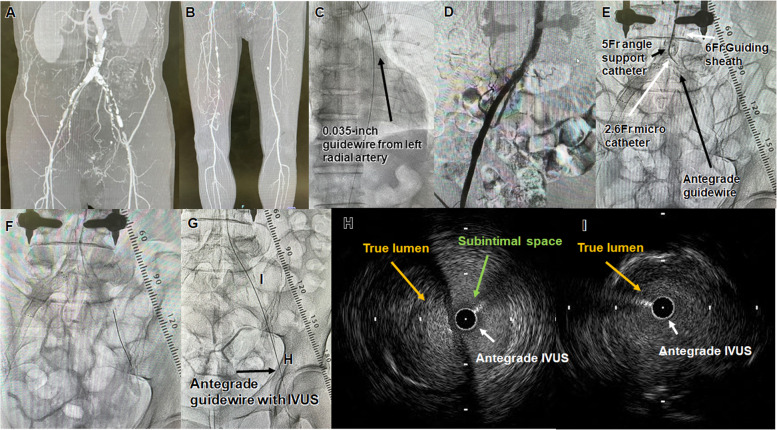
Case 1. **A, B** Preprocedural enhanced computed tomography images. **C** The transradial approach. The black arrow shows the 0.035-inch guidewire used to introduce the guiding sheath. **D** Control angiography showed a total occlusion of the left common iliac artery. **E** A 0.014-inch guidewire with a 5Fr angled support catheter and a 6Fr guiding sheath was advanced into the chronic total occlusion lesion. The white arrow on upper right shows the tip of the guiding sheath. The black arrow on upper left shows the 5Fr angled support catheter. The white arrow on lower left shows the 2.6Fr microcatheter. The black arrow on lower right shows the antegrade guidewire. **F** An antegrade guidewire was advanced to the distal external iliac artery (EIA). **G** The black arrow shows the antegrade guidewire with intravascular ultrasound (IVUS). **H** IVUS findings in the distal EIA. The white arrow shows the antegrade IVUS in the subintimal space. The yellow arrow shows the true lumen. The green arrow shows the subintimal space. **I** IVUS findings in the proximal EIA. The white arrow shows the antegrade IVUS. The yellow arrow shows the true lumen

**Fig. 2:**
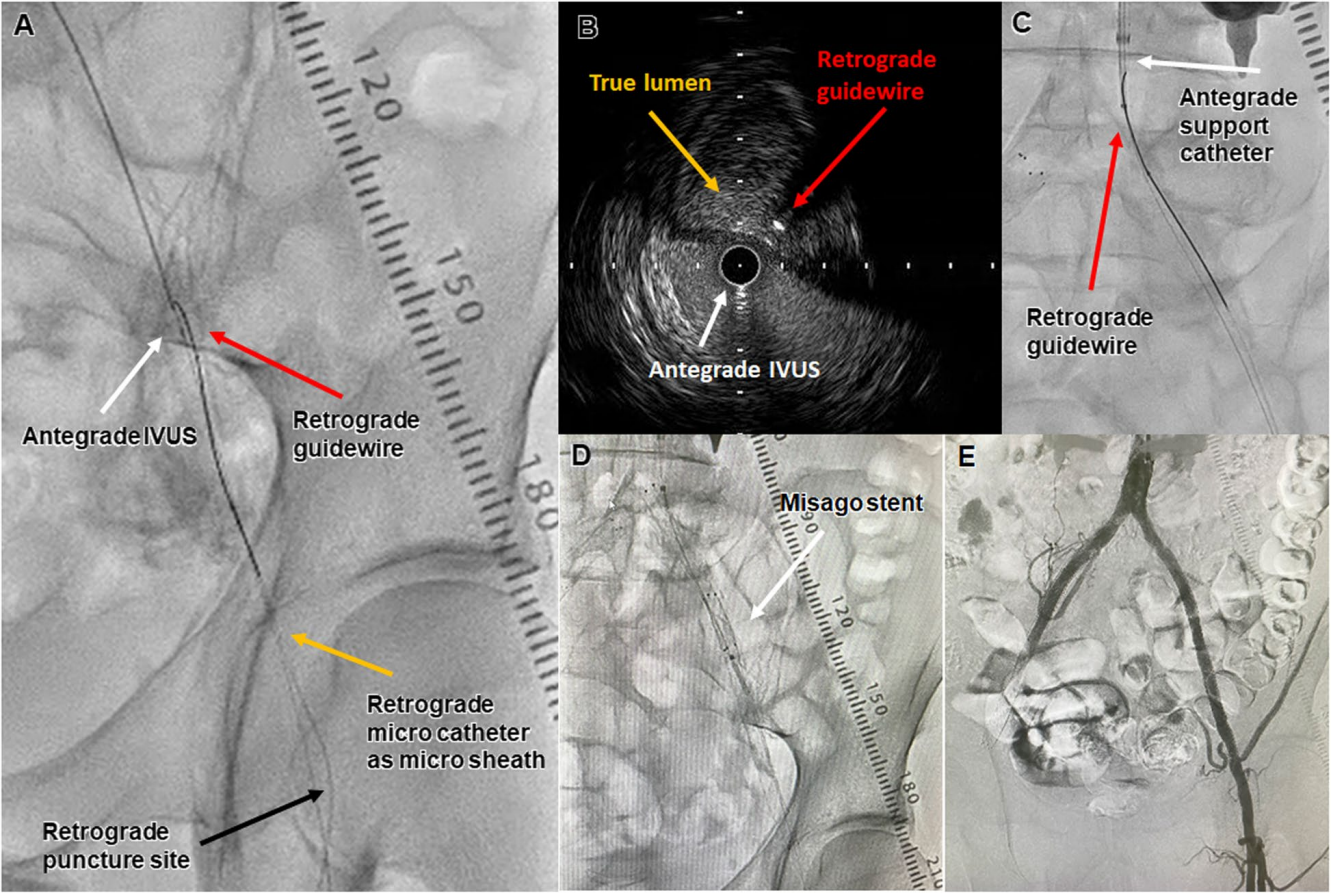
Case 1. **A** IVUS guided parallel wiring. The white arrow shows the antegrade intravascular ultrasound (IVUS). The red arrow shows the retrograde guidewire. The yellow arrow shows the retrograde microcatheter as a microsheath. The black arrow shows the retrograde puncture site. **B** IVUS findings in the distal EIA. The white arrow shows the antegrade IVUS in the subintimal space in the true lumen. The yellow arrow shows the true lumen. The red arrow shows the retrograde guidewire. **C** A retrograde guidewire was advanced into the antegrade support catheter to achieve guidewire externalization. The white arrow shows the antegrade support catheter. The red arrow shows the retrograde guidewire. **D** Two Misago stents were deployed in the iliac artery. The white arrow shows a Misago stent. **E** The final angiography showed good antegrade flow

A 71-year-old man with diabetes mellitus presented with severe claudication of both lower limbs. The patient’s ankle–brachial index (ABI) was 0.41 on the right side and 0.43 on the left side. Contrast-enhanced computed tomography (CT) showed severe stenosis of the right external iliac artery (EIA) and total occlusion of the superficial femoral artery (SFA) (Fig. 1A, B). On the left side, there was a long segment of total occlusion from the common iliac artery (CIA) ostium to the distal EIA (Fig. 1A). The right EIA was initially treated by BNS, followed by EVT to the left iliac CTO using a TRA (Fig. 1C). A 120-cm 6Fr guiding sheath (Destination Slender® guiding sheath; Terumo, Tokyo) was inserted into the left radial artery. After insertion of the guiding sheath, 5000 units of heparin were administered, and an additional 1000 units were provided every hour, throughout the procedure. Control angiography showed total occlusion of the left proximal CIA (Fig. 1D). First, a 0.014-inch guidewire (Halberd® guidewire; Asahi Intec, Aichi, Japan) and a 150-cm 2.6Fr microcatheter (Zizai® microcatheter; Terumo) with a 130-cm 5Fr support catheter (Glidecath® diagnostic catheter; Terumo) was advanced into the CIA to the EIA CTO (Fig. 1E). After the guidewire reached the distal EIA, small balloon dilation was performed, and intravascular ultrasonography (IVUS) (Navifocus WR® IVUS; Terumo) was used to observe the position of the guidewire in the CTO lesion (Fig. 1F, G). The IVUS showed that the guidewire was in the true lumen of the CIA to the middle EIA, but in the subintimal space of the distal EIA (Fig. 1H, I). The left CFA was punctured using a 21-G needle (Merit Advance® angiography needle; Merit Medical, Tokyo, Japan), and a 0.014-inch guidewire (Gladius MGES® guidewire; Asahi Intec) was inserted. Then the needle was exchanged for a 2.6Fr microcatheter (Ichibanyari PAD2® microcatheter; Kaneka, Tokyo, Japan) for a sheathless retrograde technique. Next, a 0.014-inch CTO guidewire (Halberd® guidewire; Asahi Intec) was inserted to perform the IVUS-guided parallel-wiring technique using antegrade IVUS and a retrograde guidewire (Fig. 2A). While observing the antegrade IVUS images (Fig. 2B), the retrograde guidewire was advanced into the lumen of the EIA, and guidewire externalization was finally achieved by the rendezvous technique in the CTO (Fig. 2C). After dilation with a small balloon, a 300-cm 0.014-inch guidewire (Gladius MGES® guidewire; Asahi Intec) was advanced into the SFA, and the lesion was pre-dilated with a 6.0 × 40-mm balloon (Senri® balloon; Terumo). Then, intravascular balloon occlusion using a 6.0 × 40-mm balloon in the CFA and manual compression were performed for hemostasis of the retrograde puncture site (Fig. 1E). Manual compression was also used during lesion preparation, and stenting was performed after confirming hemostasis by angiography. An 8.0 × 100-mm BNS (Misago® stent; Terumo) was deployed in the EIA, and a 10.0 × 60-mm BNS (Misago® stent; Terumo) was deployed in the just-proximal CIA (Fig. 2D, E). The final angiography showed good antegrade flow with sufficient expansion (Fig. 2F). The procedural time for EVT was 90 min, the dose of radiation exposure in terms of dose–area product was 17.0 Gycm^2^, and the amount of contrast medium was 125 ml. Postoperatively, tape was applied to the puncture site, and the patient was released from bed rest after 30 min. The urinary balloon was removed immediately after the EVT. The patient was ambulatory 30 min after the EVT and was discharged the following day. There were no complications, including postoperative radial artery (RA) occlusion, and the ABI value was 0.97 on the right side and 1.08 on the left side. Subsequent duplex ultrasonography (DUS) confirmed patency 12 months postoperatively.

### Case 2

**Fig. 3:**
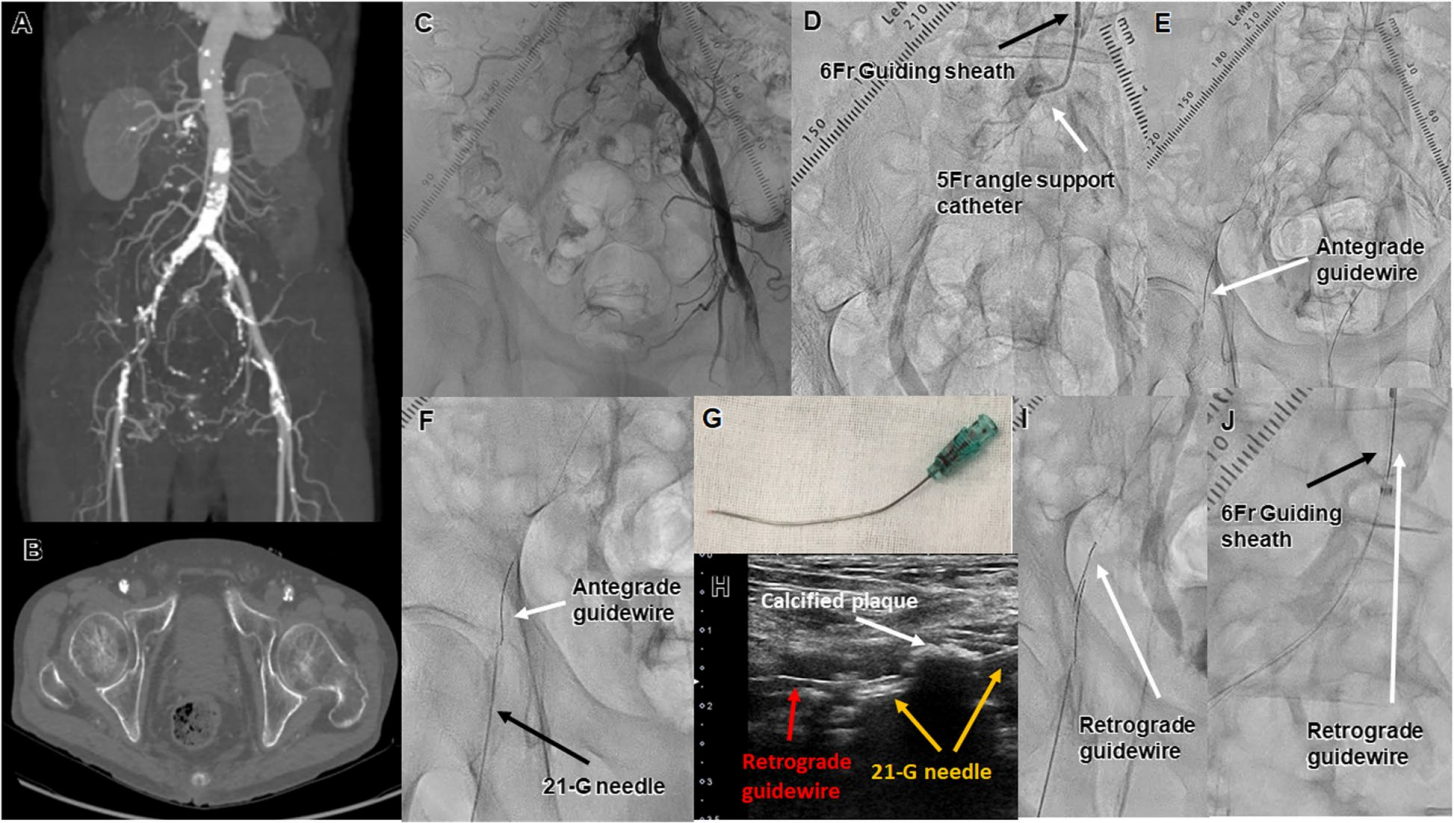
Case 2. **A, B** Preprocedural enhanced computed tomography images. **C** Control angiography showed total occlusion from the proximal aspect of the common iliac artery. **D** The black arrow shows the 6Fr guiding sheath. The red arrow shows the 5Fr angled support catheter. **E** The antegrade guidewire was advanced into the distal external iliac artery. The white arrow shows the antegrade wire. **F** The white arrow shows the antegrade wire. The black arrow shows the 21-G needle. **G** Image of a 21-G needle created with a double bend. **H** Duplex ultrasound showed that the retrograde guidewire was inside the vessel. The yellow arrow shows the 21-G needle. The white arrow shows calcified plaque. The red arrow shows the retrograde guidewire. **I** Bidirectional wiring. The white arrow shows the retrograde guidewire. **J** The retrograde guidewire was advanced into the antegrade guiding sheath to achieve guidewire externalization. The black arrow shows the antegrade 6Fr guiding sheath. The white arrow shows the retrograde guidewire

**Fig. 4:**
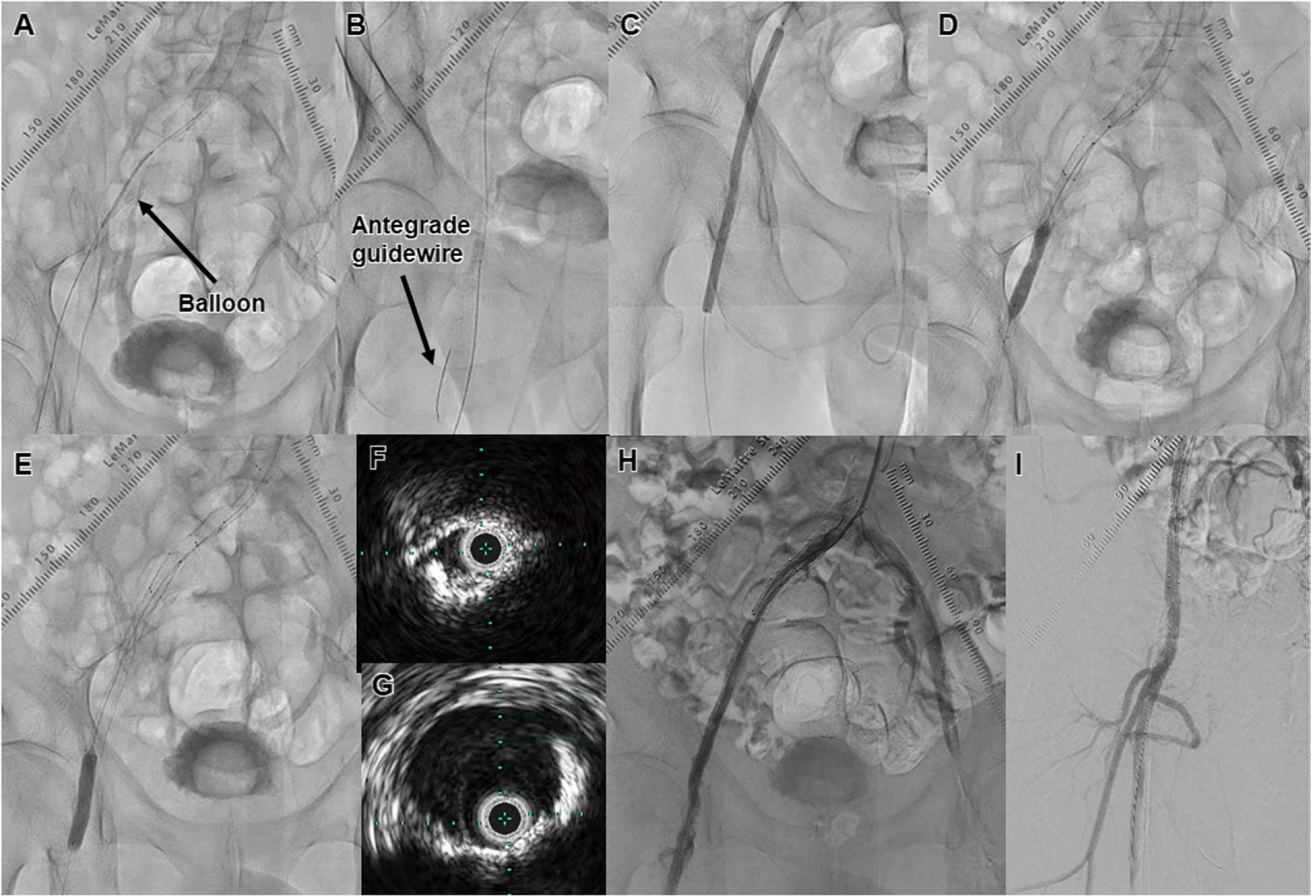
Case 2. **A** Pre-dilation was performed with a small balloon. The black arrow shows the balloon. **B** An antegrade guidewire was advanced to the superficial femoral artery. The black arrow shows the antegrade guidewire. **C** A 6.0 × 40-mm high pressure balloon was dilated with intravascular hemostasis of the retrograde puncture site. **D** Three Misago stents were deployed in the iliac artery, and post-dilation was performed. **E** A drug-coated balloon was dilated in the common femoral artery. The black arrow shows the drug-coated balloon. **F** IVUS findings after the guidewire crossing. **G** IVUS findings after the drug-coated balloon dilation. **H, I** The final angiography showed good expansion of the iliac to femoral artery, and sufficient antegrade flow

A 63-year-old man with coronary artery disease and old cerebral infarction presented with severe claudication of the right lower limb. The ABI was 0.60 on the right side. Preoperative CT showed total occlusion of the right CIA ostium to CFA, and the CFA was occluded with severe calcification (Fig. 3A, B). After confirming the absence of shaggy descending aorta by contrast-enhanced CT, EVT with the TRA was attempted. A 120-cm 6Fr guiding sheath (Destination Slender® guiding sheath; Terumo) was inserted into the left radial artery. Heparin was administered as in Case 1. Control angiography showed a total occlusion of the right just-proximal CIA to distal aspects of the CFA with calcification (Fig. 3C). First, a 0.014-inch guidewire (Gladius MGES® guidewire; Asahi Intec) and a 150-cm 2.6Fr microcatheter (Caravel® microcatheter; Asahi Intec) with a 130-cm 5Fr support catheter (Glidecath® diagnostic catheter; Terumo) was advanced into the CTO lesion (Fig. 3D). The guidewire was advanced to the distal EIA but could not be advanced further owing to the calcified occlusion of the CFA; therefore, a retrograde approach was chosen (Fig. 3E). A 21-G metal needle (Merit Advance® angiography needle; Merit Medical) that was slightly curved was advanced into the distal calcified CFA from the location of the distal lumen (Fig. 3F, G). The needle was advanced into the calcified plaque under observation from multiple directions by fluoroscopy. The needle was successfully advanced into the CTO of the distal EIA, as confirmed by DUS (Fig. 3H). A 0.014-inch guidewire (Halberd® guidewire; Asahi Intec) was inserted from behind the needle and the guidewire was advanced into the CTO lesion (Fig. 3I). After guidewire externalization by the rendezvous technique in the CTO (Fig. 3J), a 1.5 × 20-mm balloon (Coyote®; Boston Scientific, USA) and a 4.0 × 40-mm balloon (JADE®; Orbus Neich, Tokyo, Japan) were successfully passed and used to dilate the CFA to the CIA lesion (Fig. 4A). IVUS showed that the guidewire was able to completely pass through the calcified plaque (Fig. 4F). A 300-cm 0.014-inch guidewire (Gladius MGES® guidewire; Asahi Intec) was passed in the SFA direction and extended a 6.0 × 40-mm high pressure balloon (SHIDEN HP®; Kaneka) with intravascular hemostasis of the retrograde puncture site (Fig. 4B, C). After confirmation of hemostasis of the femoral artery by angiography, a 10.0 × 60-mm stent, a 9.0 × 40-mm stent, and a 8.0 × 100-mm BNS (Misago® stent; Terumo) were deployed for the CIA to the EIA, and a 6.0 × 60-mm drug-coated balloon (Lutonix RX® drug-coated balloon; BARD, AZ, USA) was deployed at the CFA lesion (Fig. 4D, E). The final angiography showed good dilation, blood flow, and puncture site hemostasis (Fig. 4G–I). The procedural time for EVT was 120 min, the dose of radiation exposure in terms of dose–area product was 17.0 Gycm^2^, and the amount of contrast medium was 150 ml. Postoperative release from bed rest was similar to that in Case 1. The patient’s clinical symptoms were ameliorated, and his ABI improved to 1.07 without RA occlusion.

## Discussion

The feasibility of EVT for complex iliac CTO using the TRA combined with a retrograde sheathless technique from the FA was demonstrated in the cases presented in this article. In this study, the weaknesses of the TRA, such as limitations of antegrade guidewire manipulation and weak pushability of crossing of devices such as balloons, were overcome by performing a minimally invasive bidirectional approach. Although effectiveness of EVT with the TRA for AI lesions has been reported, the success rate for treatment of complex lesions, such as CTO, is lower than that for stenotic lesions (Ruzsa et al., 2016; Lorenzoni et al., 2017). A combination of TRA and retrograde access was reported for treatment of complex iliac and SFA CTO lesions, where the retrograde approach used a sheath and employed a subintimal approach (Hanna et al., 2018; Hanna et al., 2016).

Use of the 6Fr compatible TRA system allows regular-size lineup for BNS placement and IVUS guidance in the iliac artery from the radial artery (Shinozaki et al., 2019). Therefore, sheath insertion is not always required, even when a retrograde approach is used. Generally, increasing distance from the RA to the iliac lesion decreases guidewire maneuverability and penetration force. To overcome this situation, a guidewire with a microcatheter and a 5Fr diagnostic catheter were used for support in both cases, which reduced deflection of the guidewire and improved maneuverability and penetration force. Use of a 5Fr angled diagnostic catheter was very helpful because it allowed us to select the entrance for CIA lesions and other types of lesions. Use of a bidirectional approach allowed guidewire externalization and dramatically improved back-up, which greatly enhanced the passage of devices such as IVUS and balloons. Because this is a bidirectional approach, the SAFARI technique or other subintimal techniques can be used when the intraluminal approach is difficult (Spinosa et al., 2005).

In Case 1, the IVUS catheter was placed in the distal EIA with an antegrade approach to permit observation, allowing relatively safe and accurate intraluminal guidewire crossing and easy passage of the wire even when a retrograde hard guidewire with a high tip load was used. In Case 2, the key was to overcome the calcified occlusion in the CFA. The standard guidewire was unable to pass through the occlusion, so a metal needle was used to directly penetrate the calcified plaque, and a guidewire was inserted into the EIA CTO, allowing passage of the guidewire. This technique was based on our previously reported BAMBOO SPEAR technique, in which a metal needle was used to directly penetrate the center of the calcified CFA (Hayakawa et al., 2021). This technique allowed us to obtain an acceptable result even in this case of a continuous occluded lesion from the iliac artery containing calcification of the CFA.

Because this was a sheathless technique with only a microcatheter inserted from the retrograde approach, hemostasis could be achieved with manual compression without problems. In both cases, combining intravascular balloon dilation and manual compression was useful for hemostasis at the retrograde puncture site. Additionally, in both cases, complete hemostasis was confirmed at the time of the final angiography, and the patients were able to walk 30 min after the application of tape at the puncture site after the procedure. This technique can be used to treat complex iliac CTO cases in a minimally invasive manner. The sheathless approach is not expected to require prolonged hemostasis; however, it is unclear whether postoperative bed rest is necessary. Further investigation is needed. In more complex cases that require covered stents, it may be advisable to use a bidirectional approach with the TRA by inserting a sheath from the retrograde approach.

There is a limitation that the procedure cannot be performed if the aorta is tortuous, bent, or affected by shaggy aorta syndrome. Preoperative CT is very important to determine the feasibility of the procedure. In our cases, initial success of the procedure and good short-term prognosis were confirmed. However, the usefulness of the method in various types of cases and its long-term results remain unclear. Another concern is that the use of covered stents is greatly limited by the TRA. It has been suggested that there are complex lesions for which a covered stent is preferable to a BNS (Mwipatayi et al., 2016), and in these cases, an additional sheath insertion of 7Fr or more from the femoral approach is necessary. There are also some limitations with the TRA for AI lesions. A long guiding sheath may not be tolerated in patients with a small-diameter RA. Additionally, when distal embolization occurs, an additional femoral approach for bailout is needed. The choice of balloons and stents with a long shaft is limited. However, if required, there are a few debulking devices with a shaft that is long enough to use with the TRA. In both of our cases, there was no radial artery occlusion after EVT. In previous reports, postoperative RA occlusion was observed in 0–6.4% of cases (Shinozaki et al., 2019; Meertens et al., 2018). In both of our cases, preprocedural DUS was used to confirm that the RA was at least 2 mm in diameter before puncture. A much larger study is required to confirm the efficacy and safety of the TRA combined with a sheathless retrograde approach.

## Conclusions

The TRA combined with a sheathless technique from the FA is an effective EVT for treatment of complex iliac CTO lesions in a less invasive manner.

## Data Availability

The datasets used and/or analyzed during the current study are available from the corresponding author on reasonable request.

## Abbreviations

ABI: Ankle–brachial index
AI: Aortoiliac
BNS: Bare nitinol stent
CFA: Common femoral artery
CIA: Common iliac artery
CT: Computed tomography
CTO: Chronic total occlusion
DUS: Duplex ultrasonography
EIA: External iliac artery
EVT: Endovascular therapy
FA: Femoral artery
IVUS: Intravascular ultrasonography
PCI: Percutaneous coronary intervention
RA: Radial artery
SFA: Superficial femoral artery
TBA: Transbrachial approach
TFA: Transfemoral approach
TRA: Transradial approach
